# A Further Contribution to the Use of the Galvano-Cautery in Pterygium Operations*Read before the Ophthalmological Section of the American Medical Association in Atlanta, May, 1896.

**Published:** 1896-09

**Authors:** Arthur G. Hobbs

**Affiliations:** Atlanta, Ga.


ATLANTA

Medical and Surgical Journal.

VOL. XIII. SEPTEMBER, 1896. No. 7.

LUTHER B. GRANDY, M.D., MANAGING EDITOR.

M. B. HUTCHINS, M.D., BUSINESS MANAGER.

ALEX. W. STIRLING, M.D., C.M., M.B. (Edin.), D.P.H. (Lond.), MANAGING EDITOR PRO TEM.

COLLABORATORS:
A. W. CALHOUN, M.D., LL.D., VIRGIL O. HARDON, M.D., FLOYD W. McRAE, M.D., AND DUNBAR ROY, M.D.

### ORIGINAL COMMUNICATIONS.

### A FURTHER CONTRIBUTION TO THE USE OF THE GALVANO-CAUTERY IN PTERYGIUM OPERATIONS.*

BY ARTHUR G. HOBBS, M.D.,

ATLANTA, GA.

Since presenting my first paper (The Treatment of Pterygia with the Galvano-Cautery) before the American Medical Association in San Francisco, two years ago, I have employed the cautery twenty-one times in pterygium proper, and twelve times in pinguecula, its usual antecedent. In my earlier contributions to this subject I pointed out that the principal object to be obtained by a pterygium operation is to prevent two raw surfaces from remaining in apposition after the operation, which necessarily results in the relapses that we too frequently see.

*Read before the Ophthalmological Section of the American Medical Association in Atlanta, May, 1896.

Why not then convert each of the raw surfaces into an eschar at once to avoid such failures, provided we are able to demonstrate practically that we can bring about that desired result?

For some years the galvano-cautery point has been resorted to in corneal ulcers, particularly in those indolent and intractable ulcers which refuse to yield to other methods of treatment. It was suggested to me a few years ago, after seeing some of the beautiful results of the cautery when applied to ulcers of the cornea, that it could, at least, produce no bad results if properly applied to the neck of a pterygium for the purpose of cutting off the nutrition of its corneal apex. I hoped it would prove a better means of severing the arterial supply to the apex as it crossed the sclero-corneal junction. This object is generally attained by the use of the knife, and various other methods have also been resorted to. The failures in pterygium operations are usually due to the re-establishment of the arterial circulation, and when this occurs the desired end of the operation fails in proportion to the re-established vessels.

The great vascularity of a pterygium, with its vessels crossing over the sclero-corneal line to supply its apex, renders it difficult to sever all the arteries by a clean cut with the knife; hence the many knife and scissors operations that have been resorted to by different operators to accomplish this end.

Each surgeon aims at one end primarily, and that is to cut off all the blood supply from the corneal apex; and secondarily, to destroy as little conjunctiva as possible. When the first end is attained, the corneal part of the pterygium has lost its direct nutrition and then depends upon the cornea proper for its sustenance; and as this meager supply proves insufficient, atrophy results and the partial, or almost complete, obliteration after a time will depend upon the activity of the absorbents. For this reason the best results are obtained in younger subjects in which, as a rule, the lymphatics are more active, as is also the contractility of the arterial coats.

In many cases when the knife is resorted to, a secondary pterygium results, in which case sufficient nutrition is supplied to the apex to perpetuate the corneal haziness even beyond its apparent margin and to greatly interfere with vision.

When the cautery section is well made I have not seen these secondary results that are often so difficult to prevent by a clean cut with the knife and scissors. In some cases, however, it may be best to combine the methods of operating, as I have done in two cases not included in this report. These two pterygia were extremely vascular and thick, hence I first used the knife in the usual way and then seared the divided edges with the cautery blade to prevent the secondary results.

The fine-pointed cautery blade, heated with a battery that can be perfectly gauged and always relied on, is applied horizontally to the narrowest portion of the growth which is near the sclero-corneal line; the application is made just before white heat is reached, and should be quickly applied and reapplied, if the tissues are not at first completely severed. If the growth is not adherent to the scleral and corneal margin, it is best to grasp it with small forceps and raise it from the sclera. When the forceps are thus used it is easier to be certain that the cautery has made complete section of all the hypertrophied conjunctival tissue.

When the corneal head is large and protruding it is advisable to make a cautery application in the same manner as in applying to a corneal ulcer. In both cases the resulting cicatrix is less—it is more transparent. This is not altogether an unknown fact, but one that has seemed never to have been brought out as prominently as its importance would suggest. For this reason one may be tempted often to resort to the cautery to reduce a large pterygium apex or a corneal ulcer, for the purpose alone of attaining this corneal transparency. The resulting eschar completely severs the vascular connection, leaving no raw surfaces in apposition, so that a re-established circulation is practically impossible; this cannot always be attained when the knife or scissors have been used.

I would have resorted to this method more frequently but for that decided objection to the cautery which we so constantly encounter, but are bound to respect. To recapitulate: The operation is quickly performed; it is followed by no bleeding; it leaves no ecchymosis; the wounds heal rapidly; there is very little pain during and after the performance; it requires no bandages and the eventual corneal haziness around the apex is slight. A weak solution of cocaine (2 per cent.) is first dropped into the conjunctival sac, then after about five minutes a stronger solution (about 10 per cent.) is applied at intervals, locally, by means of a small cotton probe, for five minutes more; in the meantime the lids are held open. By this means the toxic effects are avoided except in those subjects that are unusually susceptible to cocaine. In using cocaine, in this latter strength, I try to avoid as much as possible, by the position of the patient, its contact with the cornea. It is now a well-known fact that long-continued applications of this drug, even in weak solutions, are disastrous to an abraded cornea.

During the past two years I am inclined to restrict the use of the cautery to cases with narrow necks, those that can be raised with fine tooth forceps, and preferably to such of these as admit of a broad curved needle being introduced beneath the narrow neck at the sclero-corneal junction as it is lifted from the sclera with the forceps. One or two quick touches with the cautery knife severs the fibrous tissue down to the needle, leaving both ends seared. This procedure at the same time prevents adhesion by first intention and causes a retraction in both directions. One perpendicular stitch, to fill the conjunctival gap and the operation is complete unless it be advisable to touch the corneal apex with the cherry red point. An antiseptic dressing with vaseline and the head bound with a single round of bandage if desired suffices to lessen the friction of the lids upon the wound and to comfort during the healing process.

In three cases of double pterygia I applied the cautery on one side and made a cutting operation on the companion eye. The results, in each instance, were in favor of the cautery, that is to say, the latter method was more quickly done and with less pain, while the healing process was shorter, and the cornea (the most important consideration of all) was left with considerably less opacity than when the cutting operations were employed.

Another consideration not to be lost sight of is that should a second operation be necessary, the patient is more likely to consent to it, when the quickly performed and almost painless cauterization has been previously used than when the knife has been resorted to.

SEPT. 1.—Since the above was written I have used a broad, blunt needle, grooved on its concave as a safeguard, by first insinuating it through a small slit in the conjunctiva between the sclera and the narrow neck close to the cornea. The cautery blade then cuts through the fold down to the groove. This is at least a safer means for beginners, and I now think it is best, when the first step is practicable, as it is not if the neck is broad and adherent to the sclera.]

